# *In vitro* activity of lactone ketolide nafithromycin (WCK 4873) against *Streptococcus pneumoniae* isolates enriched with macrolide-resistance phenotype collected from mainland China

**DOI:** 10.1093/jacamr/dlac103

**Published:** 2022-10-10

**Authors:** Menglan Zhou, Lijuan Wu, Wei Kang, Yanbing Li, Ge Zhang, Jingjia Zhang, Simeng Duan, Jin Li, Tong Wang, Yingchun Xu, Yihai Gu

**Affiliations:** Department of Clinical Laboratory, State Key Laboratory of Complex Severe and Rare Diseases, Peking Union Medical College Hospital, Chinese Academy of Medical Sciences and Peking Union Medical College, Beijing, 100730, China; Department of Clinical Laboratory, Beijing Key Laboratory for Mechanisms Research and Precision Diagnosis of Invasive Fungal Diseases, Beijing 100730, China; Department of Clinical laboratory, Shenzhen Bao'an Women's and Children's Hospital, Shenzhen 518102, China; Department of Clinical Laboratory, State Key Laboratory of Complex Severe and Rare Diseases, Peking Union Medical College Hospital, Chinese Academy of Medical Sciences and Peking Union Medical College, Beijing, 100730, China; Department of Clinical Laboratory, Beijing Key Laboratory for Mechanisms Research and Precision Diagnosis of Invasive Fungal Diseases, Beijing 100730, China; Department of Clinical Laboratory, State Key Laboratory of Complex Severe and Rare Diseases, Peking Union Medical College Hospital, Chinese Academy of Medical Sciences and Peking Union Medical College, Beijing, 100730, China; Department of Clinical Laboratory, Beijing Key Laboratory for Mechanisms Research and Precision Diagnosis of Invasive Fungal Diseases, Beijing 100730, China; Department of Clinical Laboratory, State Key Laboratory of Complex Severe and Rare Diseases, Peking Union Medical College Hospital, Chinese Academy of Medical Sciences and Peking Union Medical College, Beijing, 100730, China; Department of Clinical Laboratory, Beijing Key Laboratory for Mechanisms Research and Precision Diagnosis of Invasive Fungal Diseases, Beijing 100730, China; Department of Clinical Laboratory, State Key Laboratory of Complex Severe and Rare Diseases, Peking Union Medical College Hospital, Chinese Academy of Medical Sciences and Peking Union Medical College, Beijing, 100730, China; Department of Clinical Laboratory, Beijing Key Laboratory for Mechanisms Research and Precision Diagnosis of Invasive Fungal Diseases, Beijing 100730, China; Department of Clinical Laboratory, State Key Laboratory of Complex Severe and Rare Diseases, Peking Union Medical College Hospital, Chinese Academy of Medical Sciences and Peking Union Medical College, Beijing, 100730, China; Department of Clinical Laboratory, Beijing Key Laboratory for Mechanisms Research and Precision Diagnosis of Invasive Fungal Diseases, Beijing 100730, China; Department of Clinical Laboratory, State Key Laboratory of Complex Severe and Rare Diseases, Peking Union Medical College Hospital, Chinese Academy of Medical Sciences and Peking Union Medical College, Beijing, 100730, China; Department of Clinical Laboratory, Beijing Key Laboratory for Mechanisms Research and Precision Diagnosis of Invasive Fungal Diseases, Beijing 100730, China; Department of Clinical Laboratory, State Key Laboratory of Complex Severe and Rare Diseases, Peking Union Medical College Hospital, Chinese Academy of Medical Sciences and Peking Union Medical College, Beijing, 100730, China; Department of Clinical Laboratory, Beijing Key Laboratory for Mechanisms Research and Precision Diagnosis of Invasive Fungal Diseases, Beijing 100730, China; Department of Clinical Laboratory, State Key Laboratory of Complex Severe and Rare Diseases, Peking Union Medical College Hospital, Chinese Academy of Medical Sciences and Peking Union Medical College, Beijing, 100730, China; Department of Clinical Laboratory, Beijing Key Laboratory for Mechanisms Research and Precision Diagnosis of Invasive Fungal Diseases, Beijing 100730, China; Department of Microbiology, 3201 hospital, School of Medicine, Xi'an Jiaotong University, Shaanxi 723000, China

## Abstract

**Background:**

Widespread MDR *Streptococcus pneumoniae* in China translates clinically into a substantial pneumococcal disease burden and related morbidity and mortality, particularly in the elderly and children. Nafithromycin (WCK 4873), a novel lactone ketolide class of antibiotic designed with a 3 day, once-daily regimen is highly active against resistant pneumococci and other community respiratory pathogens. It is currently in clinical development for the treatment of community-acquired bacterial pneumonia (CABP).

**Objectives:**

To determine the *in vitro* activity of nafithromycin against clinical *S*. *pneumoniae* isolates collected during 2015–21 from three hospitals in mainland China.

**Methods:**

A total of 920 clinical isolates (one isolate per patient), which predominantly with the macrolide- and clindamycin-resistant phenotype were included in this study. The MICs of nafithromycin and other antibiotics tested were determined using the reference broth microdilution method.

**Results:**

Clinical *S. pneumoniae* isolates used in this study showed high macrolide and clindamycin resistance (>95% against erythromycin and azithromycin and 80% against clindamycin) for which nafithromycin showed potent activity (MIC_50/90_; 0.03/0.06 mg/L) with 100% susceptibility at a proposed pharmacokinetics/pharmacodynamics (PK/PD) breakpoint of 0.25 mg/L. Among other classes of antibiotics tested, moxifloxacin also showed good activity while amoxicillin/clavulanate and ceftriaxone showed lower susceptibility.

**Conclusions:**

Nafithromycin exhibited therapeutically relevant *in vitro* antibacterial activity against contemporary highly resistant pneumococci collected from mainland China. This study supports the clinical development of nafithromycin for the management of CABP caused by pneumococci in China.

## Introduction

*Streptococcus pneumoniae* is a bacterium of high public health importance as it causes both non-invasive and invasive infections in the wider population, particularly in children, elderly and immune-compromised patients.^1,2^ Invasive infections include meningitis and bacteraemia while among the non-invasive infections, community-acquired bacterial pneumonia (CABP) is the most frequent. Globally, CABP incidences range between 20 and 100 per 10 000 person-years.^3^ Though CABP is a multi-aetiological infection, *S. pneumoniae* is the major causative agent,^4,5^ primarily treated with empirical antibiotics in ambulatory settings and only severe cases or instances of oral therapy failure or comorbidities require hospitalization.^6^ In the past, macrolides served as monotherapy for CABP owing to the coverage of most CABP pathogens and safety commensurate to outpatient use in more vulnerable paediatric and geriatric patients.^7^ However, the growing macrolide resistance in certain geographies, including China, has threatened the coveted ‘standard-of-care’ therapeutic status of macrolides. For instance, several reports in China have now established that macrolide/clindamycin resistance in *S. pneumoniae* isolates exceeds 80%.^8–10^ Further, these strains also exhibit reduced penicillin and ceftriaxone susceptibilities,^10,11^ thus further complicating the treatment approach. Lack of activity against β-lactams for some intracellular atypical respiratory pathogens and poor tolerability of fluoroquinolones among children and elderly patients limit their use as first-line drugs in community settings.^12^ Thus, while countries such as China are experiencing the need of new respiratory antibiotics, the paradox is that, globally, the discovery efforts in this therapeutic area have been relatively curtailed. Failure to successfully develop an outpatient and hospital-use commensurate CABP antibiotic in recent years underlines the enormous discovery and development challenges in optimising oral and IV PK, and gaining monotherapy-appropriate pathogen spectrum coverage. Such a project also throws significant challenges in terms of identifying the development candidate with a reassuring safety profile in vulnerable groups and favourable target tissue (lung) partitioning leading to a ‘compliance-friendly’ shorter-course therapy.

Nafithromycin is a novel lactone ketolide in clinical development (global Phase 2 completed, Phase 3 ongoing in India, scheduled to enter Phase 1 in China), designed to overcome all macrolide-resistance mechanisms in *S. pneumoniae* as well as offering a shorter regimen (QD × 3 days) therapy, owing to high and sustained lung concentrations.^13^ For instance, a clinical pulmonary PK study has shown that nafithromycin exposures in epithelial lining fluid (ELF) are 69 × higher and in alveolar macrophages are 2635 × higher compared with unbound concentrations in plasma.^14^ Nafithromycin’s therapeutically relevant ELF concentrations are sustained even 2 days after the last dose, which helped evolve a once-daily, 3 day regimen that was non-inferior to a 7 days regimen of moxifloxacin in a Phase 2 multinational randomized and controlled trial in CABP indication (ClinicalTrials.gov Identifier: NCT02903836). These features are attributed to the unique structural characteristics of nafithromycin involving a double-bond amidoxime core without a fluoro substitution, and a hydrophilic alkyl aryl side chain bearing chiral methyl.^15^ Recent studies have revealed a clinically relevant interesting finding of nafithromycin-mediated anti-inflammatory activity being observed in an LPS-induced acute lung injury model. In this study, nafithromycin caused inhibition of pro-inflammatory markers such as myeloperoxidase (MPO), TNF-α and IL-6, which may provide additional clinical benefits by resolving the secondary complications associated with severe pneumonia.^16^

*In vitro* activity of nafithromycin against respiratory pathogens, including *S. pneumoniae*, was earlier assessed in a global surveillance programme (barring isolates from mainland China)^17^ and also in a recent study against the *S. pneumoniae* isolates collected from India.^13^ This work describes the activity of nafithromycin against a large collection of *S. pneumoniae* isolates from mainland China, which are known to display higher macrolide/clindamycin and penicillin resistance.

## Materials and methods

### Bacterial isolates

A total of 920 clinical isolates of *S. pneumoniae* were included in the study. These isolates were collected during 2015 to 2021 from three hospitals: Peking Union Medical College Hospital (PUMCH) (*n *= 302), Shenzhen Baoan District Maternal and Child Health Care Hospital (SBH) (*n *= 329) and Shaanxi Hanzhong 3201 Hospital (SHH) (*n *= 289) in mainland China. The non-duplicate isolates were obtained from various clinical specimens (one isolate per patient) including sputum (*n* = 788), bronchoalveolar lavage fluid (*n* = 43), blood (*n* = 34), swabs (*n* = 22), secretions (*n* = 11), CSF (*n* = 9) and others (*n* = 13). The identity of the isolates was confirmed biochemically as well as by using MALDI-TOF MS. The reference strains were procured from ATCC. The isolates were stored in glycerol stocks at −80°C and inoculated on 5% sheep blood agar plates with a subsequent passage on blood agar before MIC determination.

### Antimicrobial susceptibility testing

Antimicrobial susceptibility testing of nafithromycin, erythromycin, azithromycin, clindamycin, amoxicillin/clavulanic acid, ceftriaxone and moxifloxacin was performed following EUCAST methodology using in-house-prepared 96-well broth microdilution panels that were stored at −80°C and thawed before use. Briefly, antimicrobial panels were prepared using standard powder [nafithromycin (Batch no: EWS10088) provided by WOCKHARDT, India and other antibiotics bought from National Institute for the Control of Pharmaceutical and Biological Products] and CAMHB supplemented with 5% lysed horse blood and 20 mg/L β-NAD according to EUCAST recommendation. The final inoculum was 5 × 10^5^ cfu/mL. Micro-dilution trays were incubated at 35°C in ambient air and MICs were read after 18 h of incubation as the lowest concentration of the agent that completely inhibited the visible growth. The final breakpoint for nafithromycin for *S. pneumoniae* is not assigned; hence the proportion of strains inhibited by nafithromycin at ≤0.25 mg/L (proposed PK/PD breakpoint) has been considered. Susceptibility to other antibiotics was interpreted based on EUCAST (v 11.0) criteria. *S. pneumoniae* ATCC 49619, *Staphylococcus aureus* ATCC 29213, *Enterococcus faecalis* ATCC 29212 and *Haemophilus influenzae* ATCC 49247 were used as quality control strains.

## Results

The cumulative distribution of MICs of nafithromycin and other tested antibiotics against all *S. pneumoniae* isolates included in this study is shown in Table 1. The isolate population was highly enriched with macrolide resistance as 96.5% (888/920) were non-susceptible to both erythromycin and azithromycin per EUCAST criteria. Resistance to clindamycin was 82.5%, reflecting a high proportion of macrolide-lincosamide-streptogramin resistance (MLS) phenotype among the study isolates. Regardless of the resistance mechanisms, nafithromycin demonstrated potent activity with MIC_50/90_ of 0.03/0.06 mg/L (MIC range ≤0.002 to 0.25 mg/L); 100% of isolates were inhibited at its proposed PK/PD breakpoint of 0.25 mg/L. The study isolates also revealed significant levels of resistance to β-lactam antibiotics; 47.3% resistance to amoxicillin/clavulanic acid and 22.7% resistance to ceftriaxone was observed. Expectedly, resistance to moxifloxacin was negligible (1.4%).

**Table 1.** MIC distribution of nafithromycin and other comparators tested against 920 *S. pneumoniae* isolates collected from mainland China

	Number of isolates inhibited at MIC (mg/L) (% cumulative inhibition)															EUCAST Interpretive criteria	
																	
Antibiotic	≤0.002	0.004	0.008	0.015	0.03	0.06	0.12	0.25	0.5	1	2	4	8	≥16	MIC_50/90_ (mg/L)	%S	%R
Nafithromycin	18^a^ (2.0)	80 (10.7)	141 (26.0)	147 (42.0)	152 (58.48)	318 (93.04)	60 (99.57)	4 (100)							0.03/0.06	NA	NA
Erythromycin				17^a^ (1.85)	7 (2.61)	4 (3.04)	2 (3.26)	2 (3.48)	12 (4.78)	25 (7.5)	37 (11.52)	25 (14.24)	20 (16.41)	769 (100)	>16/>16	3.5	95.2
Azithromycin				8^a^ (0.87)	2 (1.09)	13 (2.5)	3 (2.83)	3 (3.15)	1 (3.26)	7 (4.02)	13 (5.43)	8 (6.3)	22 (8.7)	840 (100)	>16/>16	3.2	96.7
Clindamycin				19^a^ (2.07)	36 (5.98)	35 (9.78)	28 (12.83)	33 (16.41)	10 (17.5)	6 (18.15)	7 (18.91)	13 (20.33)	7 (21.09)	726 (100)	>16/>16	17.5	82.5
Amoxicillin/clavulanic acid^b^				189^a^ (20.54)	41 (25)	20 (27.17)	45 (32.07)	38 (36.2)	29 (39.35)	123 (52.72)	140 (67.93)	105 (79.35)	188 (99.78)	2 (100)	1/8	39.4	47.3
Ceftriaxone				119^a^ (12.93)	64 (19.89)	50 (25.33)	36 (29.24)	67 (36.52)	52 (42.17)	191 (62.93)	132 (77.28)	143 (92.83)	66 (100)		1/4	42.17	22.7
Moxifloxacin					39^a^ (4.24)	408 (48.59)	446 (97.07)	12 (98.37)	2 (98.59)	3 (98.91)	8 (99.78)	1 (99.89)	1 (100)		0.12/0.12	98.6	1.4

%S, percentage susceptible; %R, percentage resistant; NA, not available.

a Number of isolates inhibited at lowest concentration tested.

b Clavulanic acid at fixed 2 mg/L; susceptibility interpretation was based on oral amoxicillin/clavulanic acid.

Table 2 shows the MIC distribution of nafithromycin for isolates categorized based on MDR phenotype (resistant to macrolides, clindamycin and ceftriaxone), isolate collection centre, age group and site of infection. Against MDR phenotype (*n *= 199), MIC_50_ of nafithromycin shifted minimally by just 1-fold dilution to 0.06 mg/L as compared with that of all 920 isolates. Among the three medical centres, isolates from SHH showed comparatively higher nafithromycin MICs. No difference in MIC pattern of nafithromycin was observed between isolates from paediatric and infant patients (<18 years old) versus adult patients (≥18 years old) and between isolates from invasive versus non-invasive specimens.

**Table 2.** MIC distribution of nafithromycin against *S. pneumoniae* isolates, stratified by resistance phenotype, medical centre, patient age and site of infection

Category	Number of isolates inhibited at MIC (mg/L) (% cumulative inhibition)					MIC_50/90_ (mg/L)
	≤0.015	0.03	0.06	0.12	0.25	
Resistance phenotype						
MDR phenotype (*n *= 199)	19 (9.54)	40 (29.64)	126 (92.96)	13 (99.5)	1 (100)	0.06/0.06
Hospital site						
PUMCH (*n *= 302)	166 (54.97)	75 (79.80)	47 (95.36)	14 (100)	—	≤0.015/0.06
SBH (*n *= 329)	219 (66.57)	58 (84.19)	43 (97.26)	9 (100)	—	≤0.015/0.06
SHH	1 (0.35)	19 (6.92)	228 (85.81)	37 (98.62)	4 (100)	0.06/0.12
(*n *= 289)						
Age group						
Infants and paediatrics, <18 years old (*n *537)	237 (44.13)	75 (58.1)	189 (93.3)	34 (99.63)	2 (100)	0.03/0.06
Adults	149 (38.9)	77 (59.01)	129 (92.69)	26 (99.48)	2 (100)	0.03/0.06
≥18 years (*n *= 383)						
Site of infection						
Invasive^a^ (*n *= 43)	19 (44.18)	8 (62.79)	15 (97.67)	1 (100)	—	0.03/0.06
Non-invasive (*n *=* *877)	367 (41.84)	143 (58.15)	303 (92.70)	59 (99.43)	5 (100)	0.03/0.06

a Strains isolated from blood and CSF.

Analyses of the antibiotic treatment pattern (prior to and after the isolation of the pneumococci) in the patients (from whom the study organisms were isolated) with confirmed/suspected respiratory infections showed that β-lactam/β-lactamase inhibitors and azithromycin were the commonly prescribed drugs. Among the β-lactam/β-lactamase inhibitors, piperacillin/tazobactam was the most prescribed one, followed by ticarcillin/clavulanic acid and cefoperazone/sulbactam. Several patients were administered with more than one antibiotic (Table S1, available as Supplementary data at *JAC* Online).

## Discussion

Antibiotics belonging to classes of macrolides, β-lactams and fluoroquinolones are considered as the backbone of CABP management in outpatient settings. However, substantially higher macrolide and penicillin resistance in pneumococci in China coupled with safety concerns for fluoroquinolones poses a constant treatment dilemma.^18^ Therefore, there is a need for a novel antibiotic with comprehensive activity against all CABP-causing pathogens and a safety profile suitable for a diverse patient population. Nafithromycin is being developed to address this unmet need in China and also for other regions reporting high resistance rates in community respiratory pathogens.

In the present study, against a large collection of *S. pneumoniae* isolates originating from three centres wherein >80% of isolates were resistant to both macrolides and clindamycin, nafithromycin showed potent activity (MIC_50/90_ of 0.03/0.06 mg/L) with all isolates inhibited at ≤0.25 mg/L, a PK/PD susceptible breakpoint of nafithromycin supported by Monte Carlo simulation and PTA. In a previous study, nafithromycin showed MIC_50/90_ of 0.03/0.12 mg/L against 394 macrolide- and clindamycin-resistant *S. pneumoniae* isolates collected as a part of 2014 SENTRY global surveillance.^17^ Furthermore, in a recent study conducted using *S. pneumoniae* isolated from nine medical centres located in India, nafithromycin showed potent activity with MIC_50/90_ of 0.03/0.06 mg/L, regardless of the resistance mechanisms.^13^ Thus, the activity profile of nafithromycin against *S. pneumoniae* isolates observed in this study is in agreement with previous studies. The consistent high potency demonstrated by nafithromycin against worldwide *S. pneumoniae* isolates, including those from China, suggests its ability to overcome diverse resistance mechanisms in pneumococci. For instance, in Europe, ErmB is the dominant macrolide resistance mechanism in *S. pneumoniae* while in the USA and Asia a significant proportion of macrolide resistance is also linked with MefA/E efflux pumps.^19^ Thus, results from the present study reaffirm the activity of nafithromycin against MDR phenotypes.

The potent activity of nafithromycin against macrolide-resistant *S. pneumoniae* is due to its optimized lactone ketolide structure,^15^ which enables it to overcome most common clinically relevant resistance mechanisms. It is reported that in ErmB-expressing strains that entail the methylation of domain V of 23S rRNA, the activity of ketolides is contingent to their binding affinity to another target, domain II of 23SrRNA. A favourable domain II interaction by nafithromycin was evident from its previously reported low MICs for *S. pneumoniae* that were non-susceptible to even telithromycin, indicating a high level of ErmB resistance.^17^ It is possible that this unique feature of nafithromycin helps overcome the high-level macrolide resistance in the Chinese isolates used in this study. Importantly, against this collection, the MIC_90_ of nafithromycin (0.06 mg/L) was two dilutions lower than the PK/PD breakpoint, which imparts a favourable PK/PD window, which is expected to long preserve the efficacy of nafithromycin in the face of either PK variability or future shifts in MIC_90_, if any. The advantage of short-course therapy in conjunction with the monotherapy feature associated with nafithromycin would be beneficial for Chinese CABP patients who are presently managed with fluoroquinolones (e.g. levofloxacin), for which therapy duration is for a minimum of 5 days and carries tolerability risks.^18^ Even the recently approved novel antibiotics omadacycline and lefamulin require relatively longer therapy duration for CABP indication, which might cause compliance challenges in community settings.

In conclusion, nafithromycin demonstrated a strong *in vitro* activity profile against clinically relevant *S. pneumoniae* isolates collected from mainland China. This study reinforces the value of nafithromycin clinical development due to its unique set of features: (a) low MICs against isolates collected globally and from high-resistance countries; (b) consistent activity against all the macrolide-impacting resistance mechanisms; (c) a 3 day short-course regimen; and (4) anti-inflammatory action.

## Supplementary Material

dlac103_Supplementary_Data

## Supplementary data

Table S1 is available as Supplementary data at *JAC* Online.
